# The Efficacy of Dornase Alpha (Polmozyme) in Resolving Persistent Atelectasis in Pediatric Critical Care

**DOI:** 10.7759/cureus.82655

**Published:** 2025-04-20

**Authors:** Abdulaziz Alshehri, Nada Aljassim, Ali Alharbi, Abdulqadus Aljendil, Hatem Alharbi, Shaikah Aldaraweish, Noura Alfozan, Abdulrahman Alibrahim, Bader Alotaibi, Nasser Aldhaban, Abdullelah Almutairi

**Affiliations:** 1 Respiratory Therapy, King Fahad Medical City, Riyadh, SAU; 2 Pediatric Intensive Care, King Fahad Medical City, Riyadh, SAU; 3 Pediatric Critical Care, Prince Sultan Military Medical City (PSMMC), Riyadh, SAU; 4 Pediatric Critical Care, King Fahad Medical City, Riyadh, SAU; 5 Respiratory Care, Prince Sultan Military Medical City (PSMMC), Riyadh, SAU

**Keywords:** dornase alpha, drug efficacy, king fahad medical city, modified radiological assessment score, pediatric critical care saudi, persistant atelectasis, polmozyme, pulmonary atelectasis

## Abstract

Background and objective

Dornase alpha can be considered an alternative therapy when the standard therapy fails, but evidence of nebulization through artificial airways for persistent pulmonary atelectasis is limited. The study aimed to determine the efficacy of dornase alpha nebulization on persistent atelectasis in non-cystic fibrosis patients with and without artificial airways.

Methodology

A retrospective cross-sectional study was conducted on patients admitted to the Pediatric Intensive Care Unit (PICU) of King Fahad Medical City (KFMC) between February 2020 and October 2023. The pre- and post-treatment MRAS (Modified Radiologically Assisted Score) was used to determine the treatment efficacy. The independent t-test and chi-square test were used for statistical analysis. Multivariate regression analysis was applied after estimating the propensity score to adjust baseline characteristics to reduce selection and indication bias. A value of p<0.05 was taken to indicate statistical significance.

Results

Dornase alpha was not an independent positive predictor of MRAS score improvement (B=0.326, p=0.757, Exp(B)=1.385) beyond other variables. However, the dornase group (n = 132) significantly improved the mean MRAS score by 6.08+2.69 compared to the non-dornase group's (n=143) mean MRAS score of 5.14+2.4, with a moderate effect size (Cohen's d=0.364, 95% CI: 0.126 to 0.603; p=0.03) in resolving persistent atelectasis.

Conclusion

Dornase alpha did not independently improve MRAS scores in pediatric patients with persistent atelectasis, controlling for selection/indication bias and confounding variables. The observed differences in MRAS improvement in the initial analysis were most likely due to baseline differences.

## Introduction

Atelectasis is a lung medical condition in which the lung doesn't open fully into its usual state [1], which can decrease the lung surface area, gas exchange rate and lead to pneumonia [2]. Moreover, pulmonary atelectasis symptoms can vary from a mild increase in the fraction of inspired oxygen (FiO₂) and hypoxemia to type one respiratory failure [3]. Equally important, the causes might be increased pulmonary secretion, high mucus viscosity, airway obstruction weak cough reflex and decreased diaphragmatic ability [4].

Atelectasis is often caused by sputum obstructing airways. Cystic fibrosis (CF), bronchiectasis, and bronchiolitis mucus include high levels of extracellular DNA from degenerating leucocytes and epithelial debris [5]. Lung secretions become more viscous and stickier with DNA. CF airway opening with rhDNase (recombinant human DNase) is reported to be successful [6]. DNase may also work in atelectasis-related illnesses since bronchial secretions and mucus plugs contain a lot of DNA [6].

There are mainly two types of atelectasis: obstructive and non-obstructive. Atelectasis is caused by airway obstructions such as neoplasms or mucus plugs, preventing air from reaching alveoli and triggering collapse [1]. Non-obstructive atelectasis can result from external forces like pleural effusion, pneumothorax, positive pressure accumulation, or increased alveolar surface tension, particularly in acute respiratory distress syndrome [7]. One type of atelectasis is lobar, when a lung lobe collapses completely, while the other is linear, where the collapse is button-like along the diaphragm [1]. In rounded atelectasis, a rounded mass with thickened pleura is found in chronic pleural effusion and disorders such as asbestos exposure [8]. Knowledge of such types and their causes is crucial for treatment and detection, especially before and after surgeries when atelectasis is common [3].

To address this problem, multiple solutions were invented, and one among them is dornase alpha (Pulmozyme), which is a recombinant human DNase. DNase, an enzyme produced in minute amounts in salivary glands and pancreas, reduces sputum viscosity by breaking down extracellular DNA strands. Dornase alpha, Food and Drug Administration (FDA)-approved recombinant human DNase, aids cystic fibrosis patients by reducing secretion viscosity and improving lung function [9]. The use of dornase alfa in non-cystic fibrosis patients may relieve atelectasis and secretions. Patients with critical illness or chronic lung diseases have higher DNA levels in their sputum, leading to increased viscosity. Increased sputum viscosity can cause mucus blockage, resulting in lung collapse or atelectasis. It is being used highly in non-cystic fibrosis mechanically ventilated patients with pulmonary atelectasis in nebulization form [10].

Moreover, dornase alpha can be considered as an alternative therapy when standard therapy fails with the addition of a clearance maneuver via manual resuscitator [11] and guided by the Modified Radiological Assisted Score (MRAS) [11, 12]. For instance, Meireles et al. (2021) found dornase alpha to be significantly associated with improvement in oxygenation as well as radiological findings among patients with lung injury related to ventilators [13]. However, Daiya and Sierra (2023) found no association and improvement in the function of the respiratory system, but a reduction in length of hospital stay was observed following dornase alpha dosage among non-cystic fibrosis pediatric patients, indicating its limited efficacy [14]. Therefore, a more robust study with a large sample size is needed to determine the efficacy of dornase alpha in non-cystic fibrosis pediatric patients.

The rationale for this study is that there is a scarcity of evidence on nebulization of dornase alpha through an artificial airway in mechanically ventilated non-cystic fibrosis pediatric patients with persistent pulmonary atelectasis. The study aimed to explore the efficacy of dornase alpha nebulization on persistent atelectasis in non-cystic fibrosis patients with and without an artificial airway. Moreover, the study also compared the cost-effectiveness and length of ICU stay between the two treatment modalities.

## Materials and methods

Study design

This was a retrospective study, and it was conducted on all pediatric patients admitted to the Pediatric Intensive Care Unit (PICU) of King Fahad Medical City. It was a single center-based study.

Study setting and duration

The study was carried out on pediatric patients in PICU between February 2020 and October 2023.

Recruitment of study participants

The eligibility criteria for the patients enrolled in the current study were non-cystic fibrosis pediatric patients with or without respiratory support and persistent pulmonary atelectasis from February 16, 2020 until October 10, 2023. The patients were recruited from the electronic database of King Fahad Medical City (KFMC) through the EPIC research module system after applying the proposed criteria.

Inclusion Criteria

The inclusion criteria were as follows: pediatric patients who had pulmonary atelectasis for more than 48 hours; patients who were admitted to pediatric intensive care units; and patients who received dornase alpha treatment for atelectasis.

Exclusion Criteria

The exclusion criteria were: cystic fibrosis (CF) patients; a patient who is not admitted to the pediatric intensive care unit; patient with non-persistent pulmonary atelectasis (less than 48 hours); and patients with missing or incomplete data.

Data collection procedures and tools

The data was collected from the electronic database of KFMC through the EPIC research module system. In this study, the authors monitored patients who had lung collapse during their stay in the PICU for more than 48 hours and underwent treatment with dornase alpha via nebulization. However, all patients were treated with Ventolin 2.5 mg as a standard therapy to bronchodilate and facilitate secretion removal. Also, other treatment modalities were adopted, such as manual positive pressure ventilation and chest physiotherapy. Moreover, additional therapies targeting atelectasis, like Ventolin, Atrovent, 3% hypertonic saline, and acetylcysteine, were also employed. Active diagnosis, chronic diagnosis and disease background were documented as per the PICU daily rounds note. The data was divided into two groups between pediatric patients who were treated with dornase alpha and other treatments other than dornase alpha (non-dornase alpha group). All the data mentioned were separately collected and added for both groups. Moreover, airway status, endotracheal size and level, and respiratory support (mechanical ventilator, high-flow nasal cannula or noninvasive ventilation) were monitored along with the parameters of the ventilator (e.g., a fraction of inspired oxygen (FiO₂), dynamic compliance, positive end-expiratory pressure (PEEP)), and changes were recorded within the time window of the atelectasis. Patient ICU length of stay in days, 28-day outcome, and other physiological parameters like central venous pressure and SpO₂/FiO₂ ratio were documented to determine the cost-effectiveness of the treatment.

The electronic chest radiographs were taken, and an atelectasis assessment was conducted via the Modified Radiological Assisted Score (MRAS), which is measured as follows: each lobe (including the lingula) is scored 0-3 (0=normal, 1=plate or minor infiltrate, 2=moderate atelectasis, 3=total atelectasis). The scores of the six lobes are then summed to give an 18-point score (0-18). The dynamic compliance was determined, such as the score of 0.00-1.00 (severe), 1.01-2.50 (moderate), 2.51-5.00 (mild), 5.01-10.00 (normal), and 10.01 or above (increased compliance).

The outcome measures of the study were improvement in atelectasis determined by MRAS before treatment, MRAS after treatment, and more specifically, the mean change in MRAS between both the groups. Moreover, the lung collapse resolution was computed using pre- and post-treatment MRAS scores. If the post-treatment MRAS score reduction is more than 50%, it was labeled as a complete resolution; for MRAS score changes around 50%, it was taken as a partial resolution, and other patients with MRAS score changes of 1 or 2 or less than 50% improvement in MRAS score were labeled as no resolution. The collapse resolution outcome is cross-tabulated with PEEP change to determine that the increase in PEEP score played a role in the resolution of lung collapse. The cost-effectiveness was also determined using measures such as length of hospital stay and 28-day outcomes. The lesser the stay and transfer from ICU facilities to ward, home, and discharge would be considered as cost-effective or vice versa. The data was carefully handled by the involved authors to ensure transparency and validity. The data was double-checked and entered in a main data analysis to determine the efficacy of dornase alpha.

Statistical analysis

The data was collected in an Excel (Microsoft, Redmond, WA) sheet. The statistical analysis was carried out using IBM SPSS Statistics version 28 (IBM Corp, Armonk, NY). The continuous variables such as age (days), collapse duration (days), mean central venous pressure (CVP) value, mean change MRAS, FiO₂, mean PEEP value, PEEP change, and duration of dornase dose (days), were represented as mean+SD. The categorical variables, such as gender, life status, airway status, endotracheal tube (ETT) level, affected lung lobes, dynamic lung compliance, types of respiratory support, types of atelectasis collapse, background history of patients, outcomes at the 28th day, and medications list (control group) were represented as frequency and percentage. The t-test was applied to compare MRAS scores (pre-treatment and post-treatment) between the two groups separately. The independent t-test was applied between both the groups to determine the efficacy of dornase alpha against mean MRAS change. The effect size was also determined using Cohen’s d statistics at a 95% confidence interval. Moreover, the impact of dornase alpha on physiological and respiratory parameters, such as secretion amount and color (before and after), was also determined using the chi-square test. Hence, the chi-square was also used to determine the cost-effectiveness by cross-tabbing both groups with length of hospital stay and 28th-day outcomes, lung lobe affected (category groups), dynamic compliance, and mean change in MRAS score.

Efforts to reduce bias

Owing to the retrospective study design, propensity score analysis was performed to adjust the baseline characteristics among two treatment cohorts. Propensity score analysis was applied to reduce selection bias and indication bias due to study design. Multivariate binary logistic regression analysis was performed taking MRAS improvement as the dependent variable and age, gender, airway status, PEEP changes, dynamic lung compliance, CVP changes, the ratio of FiO₂ by SpO₂, and types of atelectasis collapse and collapse duration to determine the role of potential confounders in the improvement of MRAS. A p-value <0.05 was taken to determine the statistical significance.

Ethical considerations

The study was carried out after taking approval from King Fahad Medical City (IRB-Registration Number with KACST, KSA: H-01-R-012, OHRP/NIH, USA: IRB00010471, Federal Wide Assurance NIH, USA: FVVA00018774). The anonymity and confidentiality of the patient’s data were assured. The data was handled only for analysis and research purposes for the benefit of patients. The data of the patients were de-identified before using it in research. The authors tried their best to meet the Strengthening the Reporting of Observational Studies in Epidemiology (STROBE) guidelines while reporting the findings of this cross-sectional study.

## Results

A total of 275 patients participated in the study (dornase group=132, non-dornase group=143). The pediatric patients with a mean age of 35.67 days were included. The gender distribution is almost equal, including 146 (53.6%) male infants and 129 (46.4%) female infants. The majority of the patients were alive (205, 74.54%). Of the patients, 132 patients (47.5%) were transferred to the ward and 86 (30.9%) were discharged after treatment, indicating a cost-effective treatment. The remaining respiratory and ventilatory statistics were demonstrated in Tables 1, 2.

**Table 1:** Descriptive statistics of respiratory and ventilatory characteristics CVP, central venous pressure; MRAS, Modified Radiographic Assessment Score; FiO_2_, fraction of inspired oxygen; PEEP, positive end-expiratory pressure.

Continuous variables	N	Mini	Max	Mean	SD
Age (days)	267	1	192	35.670	46.620
Collapse duration (days)	274	2	25	4.530	3.530
Mean value of CVP	210	0	26	3.650	5.850
Change in MRAS	275	0	16	5.589	2.606
FiO_2_ (%)	244	21	100	50.570	22.494
Mean value of PEEP	214	0	15	6.130	2.840
PEEP changes	189	1	3	1.920	0.743
Dornase duration of dose (days)	132	1	55	5.580	7.286

**Table 2:** Respiratory and ventilatory categorical variables. ETT, endotracheal tube; ACE, acetylcysteine; HTNS, hypertonic normal saline; RA, room air; MV, mechanical ventilation.

Categorical variable	Subcategory	Frequency (f)	Percentage (%)
Gender	Male	146	53.60%
	Female	129	46.40%
	Total	275	100%
Status	Alive	205	74.54%
	Deceased	70	25.46%
	Total	275	100%
Airway status	ETT	164	59.63%
	Tracheostomy	08	2.91%
	None	103	37.45%
	Total	275	100.0%
ETT level	Neutral	135	48.60%
	High	15	5.40%
	Deep	18	6.50%
	Right Mainstem	3	1.10%
	Tracheostomy	1	0.40%
	Total	172	61.90%
Affected lung lobes	Right Upper	52	18.70%
	Right Middle	3	1.10%
	Right Lower	8	2.90%
	Right Total	27	9.70%
	Left Upper	10	3.60%
	Left lower	17	6.10%
	Left Total	39	14.0%
	Right Upper and Middle	14	5.01%
	Right Upper and Lower	22	7.90%
	Right Middle and Lower	13	4.70%
	Bilateral Upper	16	5.80%
	Left Upper and Lower	11	4.01%
	Bilateral Lower	4	1.40%
	Right Upper and Left Lower	5	1.80%
	Right Upper Left Complete	14	5%
	Right Lower Left Upper	8	2.90%
	Total Bilateral	9	3.20%
	Total	272	97.80%
Compliance of lung	Severely Reduced Compliance (0.00-1.00)	129	46.90%
	Moderately Reduced Compliance: 1.01-2.50	63	22.70%
	Mildly Reduced Compliance: 2.51-5.00	48	17.30%
	Normal Compliance: 5.01-10.00	22	7.90%
	Increased Compliance: 10.01 and above	13	4.90%
	Total	275	100%
Types of respiratory support	HFNC	51	19.17%
	MV	167	62.78%
	O_2 _device	28	10.53%
	RA	20	7.52%
	Total	266	96.72%
Medications (before dornase or control group)	3% HTNS	107	38.50%
	ACE and 3% HTNS	29	10.40%
	Acetylcysteine	14	5%
	3% HTNS and Atrovent	18	6.50%
	Atrovent	12	4.30%
	All	4	1.40%
	None	79	28.40%
	Total	263	94.60%
Types of atelectasis collapse	Compressive	48	18.30%
	Resorptive	227	81.70%
	Total	275	100%
Outcomes at 28 days	Discharged	86	30.90%
	Transferred to ward	132	47.50%
	Expired	35	12.60%
	Still in ICU	15	4.70%

The statistical significance between the dornase group and the non-dornase group was assessed using a paired t-test pre- and post-treatment MRAS scores. A significant reduction was observed in the MRAS score among both sample pairs, with a moderate to large effect. Both treatments are effective in lowering MRAS scores after treatment. However, Pair 2 showed high significance (p<0.001) and a large effect size of Cohen's d=2.689, indicating robust clinical improvements with a stronger effect size. Moreover, the interval between both pairs was completely below zero, showing consistency and reliability of treatment efficacy (Table 3).

**Table 3:** Comparing MRAS scores (pre & post) between two groups separately. MRAS, Modified Radiologically Assisted Score

Paired Samples Statistics		Mean	N	Std. Deviation	Std. Error Mean	Correlation	t	Effect size (Cohen’s d), Point estimate [Upper, Lower value] at 95% CI	P-value (two-sided significance)
Pair 1 (non-dornase group)	Post-treatment MRAS	0.220	143	0.418	0.035	0.493	-20.286	0.581, -1.696 [-1.439,-1.951]	<0.001
	Pre-treatment MRAS	1.210	143	0.659	0.493				
Pair 2 (dornase group)	Post-treatment MRAS	2.790	132	2.328	0.203	0.580	-25.959	2.689, -2.259 [-1.936,-2.580]	<0.001
	Pre-treatment MRAS	8.860	132	3.257	0.283				

The independent t-test was applied across both groups to determine efficacy. The dornase group (n=132) showed a mean change in MRAS score of 6.08+2.69, and the non-dornase group's (n= 43) mean change in MRAS was 5.14+2.4. The mean change in the dornase group is higher than in the non-dornase group with a moderate effect size (Cohen's = 0.364 (95% CI: 0.126 to 0.603)). There is a small to moderate effect size, suggesting a statistically significant difference between the two groups (p=0.03, Table 4).

**Table 4:** Independent t-test to determine efficacy and effect size. * the sign indicates significant p-values. MRAS, Modified Radiologically Assisted Score

Independent t-test	Group	N	Mean	Std. Deviation	Standard Error Mean	Effect size (Cohen’s d), Point estimate [Upper, Lower] at 95% CI	p-value (Two-sided)
Change in MRAS	Dornase	132	6.076	2.689	0.234	2.568, 0.364 [0.603, 0.126]	0.003*
	Non-dornase	143	5.139	2.451	0.204		

The impact of dornase on physiological and respiratory parameters was found to be statistically significant after treatment, as the secretion and color showed significant improvement of near to normal physiology (p<0.05, Table 5).

**Table 5:** Comparison of groups to assess the impact on physiological and respiratory parameters. * indicate significant p-value.

Comparison		Group (dornase vs. Non-dornase)		Total	Chi-square
		Dornase	Non-dornase		p-value
Secretion Amount Before Treatment	Small Thin	10 (7.87%)	17 (13.18%)	27 (10.55%)	14.091 (0.015) *
	Small Thick	26 (20.47%)	27 (20.93%)	53 (20.70%)	
	Large Thin	2 (1.57%)	1 (0.78%)	3 (1.17%)	
	Large Thick	38 (29.92%)	23 (17.83%)	61 (23.83%)	
	Moderate Thin	8 (6.29%)	1 (0.78%)	9 (3.52%)	
	Moderate Thick	43 (33.86%)	60 (46.51%)	103 (40.23%)	
Total		127 (100%)	129 (100%)	256 (100%)	
Secretion Amount After Treatment	Small Thin	17 (13.39%)	30 (24%)	47 (18.65%)	17.445 (0.004) *
	Small Thick	29 (22.83%)	44 (35.2%)	73 (28.97%)	
	Large Thin	0 (0%)	2 (1.6%)	02 (0.79%)	
	Large Thick	11 (8.66%)	4 (3.2%)	15 (5.95%)	
	Moderate Thin	11 (8.66%)	8 (6.4%)	19 (7.54%)	
	Moderate Thick	59 (46.46%)	37 (29.6%)	96 (38.10%)	
Total		127 (100%)	125 (100%)	252 (100%)	
Secretion Color Before Treatment	White	93 (73.23%)	88 (68.75%)	181 (70.98%)	12.497 (0.014) *
	Clear	4 (3.15%)	18 (14.06%)	22 (8.63%)	
	Yellow	21 (16.54%)	16 (12.5%)	37 (14.51%)	
	Blood	4 (3.15%)	5 (3.91%)	9 (3.53%)	
	Other	5 (3.94%)	1 (0.78%)	6 (2.35%)	
Total		127 (100%)	128 (100%)	255 (100%)	
Secretion Color After Treatment	White	101 (79.53%)	88 (70.97%)	189 (75.30%)	8.836 (0.065)
	Clear	12 (9.45%)	26 (20.97%)	38 (15.14%)	
	Yellow	7 (5.51%)	4 (3.23%)	11 (4.38%)	
	Blood	1 (0.78%)	3 (2.42%)	4 (1.59%)	
	Other	6 (4.72%)	3 (2.42%)	9 (3.59%)	
Total		127 (100%)	124 (100%)	251 (100%)	

The length of hospital stay and discharge of patients were significant among both the groups. However, more patients were discharged among patients in the non-dornase group: 69 and 83 patients were transferred to the ward from critical care to post-ICU care among the dornase group pediatric patients (p<0.001). Moreover, the pediatric patients in non-dornase alpha group spent a significantly shorter duration in ICU as compared to dornase group patients within the first week, potentially reducing the cost of care (p=0.007, Table 6).

**Table 6:** Cross-tabulation to determine the cost-effectiveness of the dornase group ** indicates Fischer exact test. * sign indicates significant p-value. MRAS: Modified Radiologically Assisted Score.

Cross-tabulation		Group (Dornase vs. Non-dornase)		Total f(%)	Chi-square
		Dornase, f(%)	Non-dornase, f(%)		p-value
Change in MRAS	0.00	0 (0%)	1 (0.70%)	1 (0.36%)	23.184 (0.052)**
	1.00	2 (1.51%)	4 (2.79%)	6 (2.18%)	
	2.00	7 (5.30%)	11 (7.69%)	18 (6.54%)	
	3.00	18 (13.64%)	26 (18.18%)	44 (16%)	
	4.00	12 (9.09%)	17 (11.88%)	29 (10.54%)	
	5.00	18 (13.64%)	26 (18.18%)	44 (16%)	
	6.00	17 (12.87%)	28 (19.58%)	45 (10.36%)	
	7.00	20 (15.15%)	8 (5.60%)	28 (10.18%)	
	8.00	16 (12.12%)	5 (3.50%)	21 (7.64%)	
	9.00	14 (10.60%)	10 (6.99%)	24 (8.72%)	
	≥10.00	8 (6.06%)	7 (4.90%)	15 (5.45%)	
Total		132 (100%)	143 (100%)	275 (100%)	
Number of affected lobes	Right Upper	24 (17.91%)	28 (19.86%)	52 (18.90%)	52.133 (< 0.001)*
	Right Middle	1 (0.75%)	2 (1.42%)	3 (1.09%)	
	Right Lower	6 (4.48%)	2 (1.42%)	8 (2.91%)	
	Right Total	12 (8.96%)	15 (10.64%)	27 (9.82%)	
	Left Upper	3 (2.24%)	7 (4.96%)	10 (3.64%)	
	Left Lower	4 (2.99%)	13 (9.22%)	17 (6.18%)	
	Left Total	31 (23.13%)	8 (5.67%)	39 (14.18%)	
	Right Upper and Middle	13 (9.70%)	1 (0.71%)	14 (5.09%)	
	Right Upper and Lower	8 (5.97%)	14 (9.93%)	22 (8%)	
	Right Middle and Lower	4 (2.99%)	9 (6.38%)	13 (4.73%)	
	Bilateral Upper	4 (2.99%)	12 (8.51%)	16 (5.82%)	
	Left Upper and Lower	9 (6.72%)	2 (1.42%)	11 (4%)	
	Bilateral Lower	1 (0.75%)	3 (2.13%)	4 (1.45%)	
	Right Upper and Left Lower	3 (2.24%)	2 (1.42%)	5 (1.82%)	
	Right Upper Left Complete	5 (3.73%)	9 (6.38%)	14 (5.09%)	
	Right Lower Left Upper	2 (1.49%)	6 (4.25%)	8 (2.91%)	
	Total Bilateral	4 (2.99%)	8 (5.67%)	12 (4.36%)	
Total		134 (100%)	141 (100%)	275 (100%)	
Dynamic compliance	Severely Reduced Compliance (0.00-1.00)	18 (13.33%)	42 (30%)	60 (21.82%)	29.512 (< 0.001)*
	Moderately Reduced Compliance: 1.01 - 2.50	44 (32.59%)	28 (20%)	72 (26.18%)	
	Mildly Reduced Compliance: 2.51 - 5.00	41 (30.37%)	22 (15.71%)	63 (22.91%)	
	Normal Compliance: 5.01 - 10.00	24 (17.78%)	24 (17.14%)	48 (17.45%)	
	Increased Compliance: 10.01 and above	8 (5.93%)	24 (17.14%)	32 (11.63%)	
Total		135 (100%)	140 (100%)	275 (100%)	
ICU length of stay	1 week	31 (23.66%)	60 (44.78%)	91 (34.34%)	27.252 (0.007) *
	2 weeks	30 (22.90%)	26 (19.40%)	56 (21.13%)	
	3 weeks	14 (10.69%)	23 (17.16%)	37 (13.96%)	
	4 weeks	15 (11.45%)	7 (5.22%)	22 (8.30%)	
	5 weeks	16 (12.21%)	6 (4.48%)	22 (8.30%)	
	6 weeks	5 (3.82%)	4 (2.99%)	9 (3.40%)	
	7 weeks	4 (3.05%)	2 (1.49%)	6 (2.26%)	
	≥8 week	16 (12.21%)	6 (4.48%)	22 (8.30%)	
Total		131 (100%)	134 (100%)	265 (100%)	
28-Day outcome	Discharged	17 (12.98%)	69 (47.93%)	86 (33.27%)	47.628 (<0.001) *
	Transferred to Ward	83 (63.36%)	55 (38.19%)	132 (48%)	
	Expired	25 (19.08%)	10 (6.94%)	35 (12.92%)	
	Still in ICU	6 (4.58%)	9 (6.25%)	15 (5.45%)	
	Re-admitted to ICU	0 (0%)	1 (0.69%)	1 (0.36%)	
Total		131 (100%)	144 (100%)	275 (100%)	

Independent effects of multiple predictors on the odds of MRAS improvement in patients treated for persistent atelectasis were examined using multivariate binary logistic regression analysis. The analysis showed that the dornase group (dornase vs. non-dornase) was a statistically insignificant predictor of MRAS improvement (B=0.326, p=0.757, Exp(B)=1.385), meaning that dornase alpha was not an independent positive predictor of MRAS score improvement beyond other variables. Inclusion of the propensity score as an adjustment for selection bias was not statistically significant (B=0.369; p=0.649; Exp(B)=1.446), indicating minimal residual bias.

Male infants had twice the chances (Exp(B)=2.042, p=0.055, B=0.714) of increasing MRAS scores than female infants, and gender was marginally significant as a predictor, suggesting that gender affects treatment outcomes. MRAS improvement was not significantly affected by age (B=0.004, p=0.412, Exp(B)=1.004), airway status (p=0.545), duration of collapse, central venous pressure means trends, PEEP (B=0.075, p=0.770, Exp(B)=1.078) and dynamic compliance (B=0.053, p=0.166, Exp(B)=1.054) did not predict MRAS improvement. However, atelectasis (compressive) exhibited lower probabilities of MRAS improvement than resorptive collapse (B=-0.797, p=0.089, Exp(B)=0.450, Table 7).

**Table 7:** Multivariate binary logistic regression analysis. NA, not applicable. CVP, central venous pressure; PEEP, positive end-expiratory pressure; FiO_2, _fraction of inspired oxygen; SpO_2_, oxygen saturation Group 1=Dornase group; Gender 1=infant male patients; airway status 1=None; airway status 2=endotracheal tube; airway status 3=  tracheotomy tube; type of atelectasis collapse 1=compressive atelectasis.

Variables in the Equation	B	S.E.	Wald	df	Sig.
Group (dornase vs. non-dornase) (1)	0.326	1.051	0.096	1	1.385
Age	0.004	0.004	0.672	1	1.004
Gender (1)	0.714	0.372	3.689	1	2.042
Airway Status	NA	NA	2.134	3	NA
Airway Status (1)	-0.058	0.614	0.009	1	0.943
Airway Status (2)	-1.357	1.149	1.395	1	0.257
Airway Status (3)	-2.019	1.737	1.351	1	0.133
Collapse Duration (days)	0.043	0.048	0.792	1	1.043
Mean CVP	0.000	0.033	0.000	1	1.000
SpO_2_/FiO_2_ Ratio	-0.001	0.002	0.213	1	0.999
PEEP Changes	0.075	0.256	0.086	1	1.078
Dynamic Compliance	0.053	0.038	1.922	1	1.054
Type of Atelectasis Collapse (1)	-0.797	0.468	2.900	1	0.450
Propensity Score	0.369	0.810	0.207	1	1.446
Constant	-0.579	1.217	0.226	1	0.561

## Discussion

The study included 275 pediatric patients (132 in the dornase group and 143 in the non-dornase group) with a mean age of 35.67 days, out of which 227 (81.7%) pediatric patients experienced resorptive atelectasis at a mean duration of 4.53 days. MRAS score changes averaged 5.59+2.60, suggesting a considerable change (p=0.052). Dornase alpha was given to pediatric patients for a mean of 5.58 days. The gender distribution is equal, with the patient population made up of 146 (53.6%) male infants and 129 (46.4%) female infants. The majority of the 205 patients (74.54%) survived after treatment in both the groups. Dornase and non-dornase groups were tested for efficacy, and the dornase group (n=132) had a mean MRAS score change of 6.08+2.69, while the non-dornase group (n=143) had a MRAS score change of 5.14+2.4. The dornase group had a larger mean change than the non-dornase group with a moderate effect size (Cohen's d = 2.568, point estimation of 0.364 (95% CI: 0.126 to 0.603)), indicating a significant difference between groups (p=0.03). Dornase treatment affected physiological and respiratory parameters significantly, with improved secretion and color nearing normal physiology (p<0.05). Patient hospital stays and discharges differed significantly between the two groups. Notably, more patients in the non-dornase group showed better outcomes after 28 days (p<0.001), and the length of ICU stay was considerably shorter for pediatric patients in the non-dornase alpha group than those in the dornase group, potentially decreasing the care costs (p=0.007).

Owing to the retrospective study design, multivariate regression analysis was performed after propensity score analysis of patients treated with dornase and not with dornase to reduce selection and indication bias. The multivariate analysis results suggested that dornase alpha does not independently improve MRAS scores in patients with persistent atelectasis, adjusting for potential confounders. Outcome data available show that gender, as well as the degree of atelectasis collapse, may play a marginal role. Clinical and demographic variables did not show other significant associations with MRAS improvement (p>0.05). The implication of these findings reinforces the necessity of further research, possibly with prospective study designs, to validate the role of dornase alpha and to reduce the probability of potential residual biases.

Dornase alpha showed statistical significance in improving MRAS and respiratory function with a moderate effect (Cohen's d = 0.364 (95% CI: 0.126 to 0.603)) with a p-value equal to 0.03 among pediatric non-cystic fibrosis patients suffering from persistent atelectasis. The results are consistent with the study of Meireles et al. (2021) [13]. The authors found in their case reports of two cases that a one-year-old boy and a 17-year-old girl showed significant improvement while treated with dornase alpha. The authors also found similar improvement in radiological findings and respiratory parameters, for example, change in secretion color and amount and change in MRAS score after treatment (p<0.05) [13]. Similarly, Özyazıcı et al. (2022) also observed significant clinical efficacy and improvement in respiratory function with a resolution of atelectasis in a 12-day-old male infant when treated with dornase alpha. The infant was admitted after one day of heart surgery due to a complaint of atelectasis; the treatment was successful with dornase alpha [15]. Llaque-Quiroz et al. (2023), in their retrospective cross-sectional study, compared the efficacy of dornase alpha with fibro-bronchoscopy among children suffering from congenital heart disease. The authors found both treatments as statistically significant and effective in reducing atelectasis assessment scores with reporting of similar side effects among both treatment options. However, the authors also claimed that dornase is considered a suitable alternative therapeutic option against more invasive procedures [16]. The results are aligned with the findings of this present study. If critically analyzed, most of the evidence is based on less robust research designs such as case reports and retrospective observational studies. Therefore, more robust study designs are recommended in future research to validate the findings. However, the results of this research were based on the effect size estimation of Cohen's d-test to determine the consistency and validity of the results. Cohen's d value of 2.568 with a statistically significant p-value (p<0.05) showed there is consistency and validity of results, which ensured the authenticity of the study, but a more robust randomized controlled trial is needed get either a large effect size or poised contrasting point to challenge the evidence.

On the contrary, the existing literature also posed contrasting points of view on the aspect of clinical efficacy of dornase alpha in non-cystic fibrosis pediatric patients with atelectasis. Daiya and Sierra (2023), in their single-center retrospective study, found no statistically significant association of dornase alpha in improving respiratory parameters, observing no significant change in respiratory parameters such as FiO₂ or oxygenation indexes [14]. The results are a complete contrast to what we observed in our single-center retrospective study at King Fahad Medical City among pediatric patients. While critically evaluating and comparing both studies, there are some questions that need to be considered because Daiya et al. (2023) compare these outcomes only after 24 hours of dornase administration, but this study considers these outcome parameters for up to 28 days after the administration of dornase alpha, indicating more authenticity in the results. The contrasting results can be addressed only by prospective cohort studies with sufficient follow-up of outcomes. Therefore, future researchers are encouraged to focus on this critical point of discussion.

In a literature review, Thornby et al. (2014) found in their review, which included eight clinical trials and 12 case reports between 1946 and 2014 from Medline/PubMed, that the synthesized evidence showed that dornase alpha is a viable and labeled treatment option for treating non-cystic fibrosis pediatric patients with pulmonary atelectasis after the failure of conventional therapy. The review indicated dornase alpha as a viable and effective therapeutic option when conventional therapeutics failed due to limited post-conventional therapy options available [17]. This point raises the discussion of whether it is only used after conventional treatment failure or can be used as a choice of treatment. This study did not answer this point, but the authors of the current study compared both treatments and found statistically significant and better effects in dornase-treated groups. However, this is a retrospective research design, which may not be sufficient to decide on the choice of treatment. Therefore, a randomized controlled trial is needed to determine the choice of treatment option, considering both treatment options as equally comparable and ensuring balanced baseline characteristics among both groups.

In this current study, the length of ICU stays and discharges differed significantly. Notably, more patients in the non-dornase group showed better outcomes after 28 days (p<0.001), and the length of ICU stay was considerably shorter for pediatric patients in the non-dornase alpha group than in the dornase group, potentially decreasing care costs (p=0.007). Similarly, Vettleson et al. (2023) applied dornase alpha in pediatric bronchitis patients and found that these patients spend more time on ventilation and extended PICU time as compared to standard treatment group patients [18]. Porter et al. (2024), in a randomized controlled trial, compared dornase alpha with the best available care group (conventional treatment). They found no significant difference between the length of ICU stay and time on oxygenation between both groups (p>0.05) [19]. These results showed that dornase alpha may be beneficial, but their results on reducing length of ICU stays, oxygenation time, and overall cost-effectiveness are still inconclusive. But the overall impact is that non-dornase alpha treatment options are more cost-effective as compared to dornase alpha treatment. The results show conflicting evidence. Future studies are recommended to explore more cost-related factors to determine the exact cost of the treatment options and more research is also needed on determining the cost of dornase alpha for non-cystic fibrosis pediatric patients with pulmonary fibrosis because current literature is mostly presented in context to the COVID-19 or systemic inflammation-related conditions.

The study is retrospective in design, which may inherently involve selection or information bias although data is double-checked and handled with care to avoid misinformation that may induce publication bias. The standardized tests and techniques were used in the data collection and analysis. The modified radiological assessment score was used to determine the treatment effectiveness, which is trustworthy and standardized. The respiratory and physiological parameters were also considered in the analysis to observe further improvement in respiratory function after dornase alpha treatment. Some of the data is also missing, which may induce results reporting bias, but the data was excluded to prevent information bias.

The current literature is mainly based on retrospective observational and case reports; there is a need for a more robust randomized controlled trial to determine a large effect size to validate the findings. Moreover, prospective cohort or randomized controlled trial studies with sufficient follow-up of outcomes are also needed to get a comprehensively long-term, at least 28-day outcome data for dornase and conventional treatments, which is one of the strengths of this study. The randomized controlled trial also helps to determine the choice of treatment option while considering both treatment options as equally parallel or comparable, ensuring baseline characteristics balance among both the groups to consider either of the treatments as a treatment of choice.

## Conclusions

Dornase alpha did not independently increase MRAS scores in pediatric patients with persistent atelectasis, controlling for selection, indication bias, and confounding variables. The observed differences in MRAS improvement in the initial analysis were most likely due to baseline differences between the dornase and non-dornase groups. Male infants had twice the chances of increasing MRAS scores than female infants, suggesting marginally significant predictors. MRAS improvement was not significantly affected by age, airway status, duration of collapse, central venous pressure mean trends, PEEP changes, or dynamic compliance; they did not predict MRAS improvement. These results highlight the need for studies to reduce selection bias and confounding to achieve valid results. Dornase alpha may be a promising clinical treatment option due to its moderate effect size; however, its cost is higher than conventional therapy. But without a controlled, balanced, prospective longitudinal study, it is difficult to evaluate the efficacy or cost implications of dornase alpha in resolving persistent atelectasis in pediatric patients.
